# Theory of Mind Indexes the Broader Autism Phenotype in Siblings of Children with Autism at School Age

**DOI:** 10.1155/2016/6309189

**Published:** 2016-01-10

**Authors:** Tawny Tsang, Kristen Gillespie-Lynch, Ted Hutman

**Affiliations:** ^1^Department of Psychology, University of California, Los Angeles, CA 90024, USA; ^2^Department of Psychology, College of Staten Island and The Graduate Center, CUNY, New York, NY 10314, USA; ^3^Department of Psychiatry & Bio-Behavioral Sciences, David Geffen School of Medicine at UCLA, University of California, Los Angeles, CA 90024, USA

## Abstract

Subclinical variants of the social-communicative challenges and rigidity that define autism spectrum disorder (ASD) are known as the broader autism phenotype (BAP). The BAP has been conceptualized categorically (as specific to a subset of relatives of individuals with ASD) and dimensionally (as continuously distributed within the general population). The current study examined the compatibility of these two approaches by assessing associations among autism symptoms and social-communicative skills in young school-age children with ASD, children who have a sibling with ASD, and children without a sibling with ASD. Autism symptoms were associated with reduced Theory of Mind (ToM), adaptive skills, cognitive empathy, and language skills across the full sample. Reduced ToM was a core aspect of the BAP in the current sample regardless of whether the BAP was defined categorically (in terms of siblings of children with ASD who exhibited atypical developmental) or dimensionally (in terms of associations with autism symptoms across the entire sample). Early language skills predicted school-age ToM. Findings support the compatibility of categorical and dimensional approaches to the BAP, highlight reduced ToM as a core aspect of the school-age BAP, and suggest that narrative-based approaches to promoting ToM may be beneficial for siblings of children with ASD.

## 1. Introduction

Subclinical characteristics associated with autism, such as difficulties with social communication and rigidity, are continuously distributed in the general population and are particularly common among relatives of people with autism [1–5]. Collectively, these traits are referred to as the “broader autism phenotype” (BAP). Investigations of the BAP range from operationalizing it as a categorical entity restricted to a subgroup of relatives of people with autism who show elevated social-communicative impairments and/or restricted behaviors [3, 6–9] to exploring it as a dimensional phenomenon associated with a range of mental health issues and personality-related characteristics in the general population [10–13].

These different conceptualizations of the BAP often use distinct measures and sampling techniques (e.g., [14, 15]). Few studies have attempted to bridge categorical and dimensional perspectives of the BAP by determining whether specific challenges present among individuals with autism are also present among some relatives of individuals with autism, and if such challenges are continuously associated with autistic-like traits across a general population sample (see [16] for a rare example of a study that melded both approaches). Studies that include both categorical and dimensional approaches to the BAP are essential for demonstrating that they represent a common construct. The current study examines the compatibility of these two approaches by assessing autistic-like traits and social-communicative skills among young children with autism, children who have a sibling with autism, and children without a sibling with autism. This study is grounded in the idea that the BAP is akin to the “‘duality' of light, a phenomenon with both wave- and particle-like properties” [17, p. 529].

### 1.1. Relations between the BAP and Autism

Autistic-like social-communicative atypicalities and rigid behaviors may share common genetic underpinnings with autism spectrum disorder (ASD; [2]). Heightened levels of autistic-like traits are more likely to be present in parents [13, 18] and siblings of children with ASD [3, 6, 7, 19–22] than in individuals with no family history of ASD. The incidence of subclinical autistic-like traits is also greater for families with multiple instances of ASD relative to simplex families [18, 23–27]. The BAP may reflect an intermediate expression of genetic susceptibility to ASD, similar to the proposed intermediate phenotypes associated with other highly heritable psychiatric conditions, like schizophrenia and bipolar disorder (e.g., [28, 29]). Better characterization of the BAP may help identify mechanisms underlying ASD.

Variations in the nature and magnitude of phenotypic expression of the BAP observed across studies suggest heterogeneity in the aggregation of autistic-like characteristics (for review, see [4]). Although this heterogeneity may partially reflect differences in how the BAP is conceptualized, heterogeneity remains as much of a challenge for the BAP as it is for ASD itself. While the BAP is defined by milder variants of the behavioral challenges associated with ASD, it does not necessarily entail functional impairment [30] and may even be associated with giftedness (e.g., [31]). Furthermore, there is currently no agreed upon gold standard measure like the Autism Diagnostic Observation Scale [32] to quantify the extent to which an individual manifests BAP features. Instead, several instruments have been adapted or developed to characterize the BAP in family members (for a review, see [15]) and/or the general population (e.g., [14]).

### 1.2. Characterization of the BAP

An emerging body of research examines the BAP dimensionally by administering self-report measures to “general population” samples (commonly consisting of convenience samples of college students). These studies have revealed associations between heightened autistic-like traits and reduced ToM and enhanced perceptual skills [33], difficulty interpreting emotions [12, 13, 16, 34], and social challenges [11, 35].

These dimensional approaches to characterizing the BAP are believed to build upon earlier research evaluating the BAP categorically among relatives of individuals with ASD. In the first study to provide clear evidence of the BAP, Folstein and Rutter [36] found that monozygotic twins of individuals with ASD who did not themselves have ASD exhibited cognitive, linguistic, and/or social challenges. Subsequent researchers have evaluated the BAP categorically by comparing relatives of individuals with ASD more generally to those without a relative on the spectrum. For example, comparisons of parents who did or did not have a child with ASD revealed that decreased Theory of Mind (ToM; [37]) and enhanced perceptual skills may be characteristics of the BAP [38]. Other researchers have used cut-off scores on structured interviews, surveys, and/or observational measures to identify subgroups of relatives of individuals with ASD who exhibit heightened autistic-like traits [3, 6, 7, 39, 40]. A subset of parents of children with ASD who were classified as aloof based on interviews exhibited difficulties with ToM and emotion recognition relative to those who were not classified as aloof and control parents [8, 39].

In addition to subclinical levels of core autistic-like traits, studies of relatives have also revealed that developmental language atypicalities (including spelling and reading difficulties; [6]), anxiety and mood disorders, difficulties with executive functioning, and cognitive challenges are present in a subset of relatives of individuals with ASD (reviewed in [41]). However, a number of other studies have revealed no evidence that cognitive and/or language difficulties are elevated among relatives of individuals with ASD (reviewed in [42]).

### 1.3. The BAP at School Age

Among younger siblings of children with ASD who have reached school-age (the focus of this report), the BAP has been characterized broadly based on risk group [43–46] or more narrowly by focusing on a subset of siblings who exhibit heightened autistic-like symptoms and/or atypicalities in language, cognition, and academic skills [42, 47, 48]. Indeed, there is no consensus in the field regarding inclusionary criteria for BAP classification among younger siblings of children with ASD (reviewed by [49]).

The small body of emerging research focusing on the school-age outcomes of younger siblings has revealed limited evidence of the BAP at school-age. For instance, Warren and colleagues [46] compared 39 younger siblings of children with ASD (sibs-ASD) to 22 younger siblings of typically developing children (sibs-TD) at 5 years of age and found no significant differences in IQ, language, social symptoms, or behavior problems. However, reduced executive functioning and heightened levels of restricted interests/behaviors were observed among sibs-ASD. Given that sibs-ASD had exhibited reduced joint attention relative to sibs-TD as toddlers [50], the researchers surmised that sibs-ASD may have demonstrated developmental resilience by overcoming initial challenges.

Evidence suggestive of resilience was also obtained by Gamliel and colleagues [42] when they compared 37 sibs-ASD to 47 sibs-TD at multiple time points between 4 months and 7 years of age. They found that almost a third of the sibs-ASD exhibited linguistic and cognitive impairments on standardized measures at some point in development. However, most of the sibs-ASD had caught up to their peers—often without intervention—by 54 months of age, although some of them still exhibited language difficulties. Notably, the proportion of sibs-ASD who exhibited the BAP at 7 years was substantially higher than it had been between 4 and 54 months of age. Cognitive, language, and/or academic difficulties reemerged at 7 years of age and were apparent in 40% of the sibs-ASD (relative to 16% of the sibs-TD) according to parent reports and/or direct tests. The authors suggested that BAP related issues may (re)emerge as children are faced with increasingly challenging academic and social environments.

In contrast, a different research group found no evidence of impairments in expressive/receptive language, pragmatic language, or reading among a slightly older sample (8–11 years old) of 18 sibs-ASD relative to national norms [43]. Similarly, comparisons of 31 sibs-ASD and 23 sibs-TD at approximately 7.5 years of age (including some of the participants who will be described in this report) revealed no evidence of pragmatic or structural language impairments associated with the BAP [44].

However, group-level comparisons of sibs-ASD to sibs-TD may obscure atypicalities among a subset of relatives. Indeed, a recent cross-institutional study (including some of the participants described in this report) compared a subset of sibs-ASD, who were not diagnosed with ASD but who were identified as clinically concerning, to typically developing children at early school age [51]. Clinical concerns included elevated autistic-like symptoms, ADHD symptoms, speech-language challenges, anxiety/mood problems, and learning difficulties. Clinically concerning sibs-ASD had higher parent-reported autism symptoms and psychopathology and lower language skills than typically developing sibs-ASD and sibs-TD, who did not differ from one another.

In the current report, we define the BAP categorically as clinical concerns in individuals who do not meet criteria for ASD. This operational definition is consistent with prior school-age research wherein the BAP was defined in terms of a range of concerns rather than by focusing only on participants with elevated autistic-like symptoms (e.g., [42]). Miller and colleagues viewed the BAP as specific to the subset of clinically concerning children who exhibited heightened autism symptoms. However, they acknowledged that mood, language, learning, and attention difficulties are common among relatives of individuals with ASD and stated that research is needed to determine how to define the BAP among younger siblings. A primary aim of this report is to determine whether clinically concerning non-ASD outcomes are an appropriate definition of the BAP by evaluating if difficulties that are elevated among clinically concerning participants are dimensionally associated with autistic-like symptoms across the entire sample.

### 1.4. Is ToM an Aspect of the BAP at School Age?

Given that the majority of studies examining the BAP at school age have focused on linguistic, cognitive, and academic outcomes, we examine potential social-cognitive contributors to the BAP at school age. The school-age time point is a developmentally appropriate period to assess explicit ToM reasoning because evidence of rudimentary ToM (i.e., passing false-belief tasks) emerges by 4 years of age among typically developing children and becomes more sophisticated over time [52]. A subset of the children in the studies conducted by Gamliel and colleagues (24 sibs-ASD and 24 sibs-TD) participated in ToM tasks (the false belief and strange stories tasks) at 54 months of age [45]. After observing no differences in ToM between sibs-ASD and sibs-TD, the researchers concluded that sibs-ASD show resilience in terms of ToM abilities. However, this finding contrasts with research demonstrating reduced ToM among three-year-old siblings of children with ASD [53], slightly older sibs-ASD [54], and parents of children with ASD [37, 38]. Impaired ToM was also apparent among a subset of parents of children with ASD who were classified as socially aloof (e.g., [8, 39]).

Nevertheless, Shaked and colleagues' lack of evidence that reduced ToM is an aspect of the BAP at school age is consistent with null effects obtained in a study comparing 18 siblings of children with ASD (between 8 and 18 years of age) to 18 siblings of children with a learning disability [55], although the authors of the latter study concluded that their study was insufficiently powered to detect an effect if one were present. Similarly, dimensional associations between autistic-like traits and challenges with mentalizing have been demonstrated in the general population [33], but such associations are not always observed (e.g., [56]).

Contradictory evidence concerning whether ToM is an aspect of the BAP may reflect an often unexamined factor in the aforementioned studies, that is, variation in language skills. Language is concurrently associated with performance on ToM tasks for people with and without ASD (e.g., [57–59]). Language ability also predicts subsequent ToM development among children with and without ASD [60–62]. Therefore, a secondary aim of the current study is to examine associations between language (at 3 years of age and school age) and school-age ToM.

In the current study, we utilize both a behavioral measure of ToM and a parent-report measure of cognitive empathy (i.e., understanding of others' perspectives) and affective empathy (i.e., emotional responsiveness to others' emotions). Although ToM and cognitive empathy are often described as synonymous (e.g., [63]), a behavioral measure of ToM (the Eyes Test) was unrelated to cognitive and affective empathy among adults in the general population [64]. In contrast, behavioral ToM was associated with* both* cognitive and affective empathy among individuals with ASD and parents of children with ASD, who exhibited reduced cognitive and affective empathy and ToM relative to controls. Given variable associations between behavioral ToM and cognitive empathy, we evaluate both in the current study. We also include a behavioral proxy (attention to an examiner in distress) and a parent-report measure of affective empathy. However, we do not expect affective empathy to be an aspect of the BAP based on theoretical work suggesting that affective empathy may be unimpaired or enhanced in ASD [65].

### 1.5. Study Aims

A primary aim of the current study is to evaluate the compatibility of findings derived from a categorical and a dimensional approach to defining the BAP at school age. Given the broad range of variables that have been associated with the BAP in prior research, we examine categorical and dimensional associations between the BAP and a range of social-cognitive and social-affective characteristics. Study measures include observational and parent-report measures of autistic-like traits (assessed by severity scores on the ADOS), ToM, language, nonverbal IQ, affective and cognitive empathy, responses to others' distress, and adaptive behaviors. We first evaluate the extent to which these skills differ across groups to identify atypicalities apparent among younger siblings identified as clinically concerning (a categorical analysis) and then examined dimensional interrelationships between autistic-like symptoms and social-communicative variables. We then examine whether language development (at 3 years and at school age) is associated with school-age social-cognitive challenges.

Participants took part in a larger longitudinal study employing an infant-sibling design and thus the data reflect a wide range of social (dis)ability. The study seeks to determine whether a high level of shared variance among measures reflects a common underlying social-communicative deficit in the BAP, or whether individual measures uniquely account for heterogeneity in the BAP. Given the scope of skills examined, this study also examines the utility of converging measurement tools in clinical settings. The current investigation is not exhaustive but focuses on behavioral impairments in the social-affective and social-cognitive domain. In sum, the current study uses categorical and dimensional approaches to identify social characteristics associated with the BAP among young school-age children and to determine if early language predicts social challenges at school age.

## 2. Methods

### 2.1. Participants

Enrollment criteria, recruitment strategy, and a large proportion of the sample itself have been described elsewhere [44]. Infant siblings of children with autism (HR group) and low-risk (LR) controls were seen at 5 time points from infancy to toddlerhood (6, 12, 18, 24, and 36 months) and at school-age (M = 5.74 years, SD = 0.69 years). This study focuses on the latest time point in the longitudinal study and includes the subset of children who were seen at the school-age visit (*n* = 69).

Clinical psychologists observed participants at the school-age visit to determine whether they met diagnostic criteria for an ASD upon reviewing reports provided by the ADOS, the ADI-R, and the* Social Communication Questionnaire *(SCQ; [66]). Participants were classified into 4 groups for statistical comparisons (see Table 1 for sample characterization): (a) “ASD”: HR participants who met DSM-IV criteria for ASD (*n* = 9) and LR participants who received an ASD diagnosis (*n* = 2); (b) “HR-Other”: HR participants who showed other developmental concerns (e.g., heightened autistic traits, language, learning and/or cognitive delays, attention deficits, and anxiety/mood difficulties; see [51]) based on clinical judgment but did not meet clinical criteria for ASD (*n* = 12); (c) “HR-Typical”: HR participants who exhibited typical development according to the ADOS, DAS-II, CELF-4, and VABS-II (HR-TD; *n* = 24); (d) “LR-Typical”: LR participants who exhibited typical development using the aforementioned criteria (LR-TD; *n* = 22). Two additional LR participants were judged to exhibit developmental concerns and were excluded from analyses.

### 2.2. Measures

#### 2.2.1. Autism Diagnostic Observation Schedule (ADOS, [32])

The ADOS is a semistructured play-based observational measure that is designed to elicit autism symptomatology. The appropriate ADOS module (2 or 3) was administered based on participant's age and language abilities as prescribed by the authors of the measure. Symptom severity was compared across modules using the ADOS Calibrated Severity Score (ADOS-CSS; [67]), where higher scores denote more severe social disability and/or restricted interests and repetitive behaviors. ADOS severity scores exhibit more stability over time than raw scores and have therefore been recommended as a more valid measure of the severity of autistic traits [68].

#### 2.2.2. Differential Ability Scales, 2nd Edition (DAS; [69])

The DAS is a cognitive assessment measuring verbal, nonverbal, and spatial abilities. Here we focus on nonverbal cognitive ability (NVIQ), which was derived from the Nonverbal Reasoning and Spatial Abilities Standard Score.

#### 2.2.3. Clinical Evaluation of Language Fundamentals-4 (CELF-4; [70])

The CELF-4 is a standardized behavioral assessment of general language abilities in children 5 to 21 years of age. The Core Language Standard Score (CLS) is calculated based on performance on four subtests measuring receptive and expressive language skills fundamental to effective communication (e.g., semantics, syntax, morphology, auditory memory, and phonological awareness).

#### 2.2.4. Mullen Scales of Early Learning [71]

The Mullen is a standardized play-based assessment that measures nonverbal and verbal cognitive skills. This assessment was administered at the 36-month visit. Here we focus on the age-normed verbal score, which is computed from the expressive and receptive subscales.

#### 2.2.5. Griffith Empathy Measure (GEM; [72])

The GEM is a parent-report measure used to quantify individual differences in cognitive and affective empathy. Cognitive empathy refers to the ability to take another person's perspective by decoding emotions and situational cues. The affective component of empathy refers to showing appropriate or congruent affective responses to other person's situation. Cognitive and affective empathy scores are derived from parent ratings of 23 questions on a 9-point Likert scale (−4 to 4).

#### 2.2.6. Observational Empathy Task

This study included an observational measure of attentional and affective response to an examiner's display of distress during a play interaction. The measure has also been employed in previous research in our lab (e.g., [73, 74]). An examiner and the child took turns playing with a toy xylophone while the parent sat near the child. After the examiner determined that the child was focused on the play interaction, the examiner feigned injury by pretending to hit her hand with the toy mallet. At that point, the examiner withdrew from the play interaction and focused on her “hurt” hand for 15 seconds before reengaging the child and assuring the child that she felt fine. The child and examiner were filmed and the child's responses to the examiner's distress were coded offline (see [73] for additional details). Looking time at the examiner or parent is considered a proxy for attentiveness to the examiner's distress.

#### 2.2.7. Theory of Mind Task (ToM)

ToM indexes the understanding of others' mental states and represents a critical social cognitive skill for social adaptation. The task presented here is derived from Wellman and Liu [52] and assesses multiple milestones in social cognitive development. This approach has been validated in a group of children with ASD [75]. Participants were verbally presented with four brief scenarios and asked questions about each scenario that tapped into increasingly sophisticated aspects of understanding other people's mental states. Control questions were also posed to confirm comprehension of the scenario. The aspects of ToM that were evaluated proceeded in the following order: recognizing diverse desires, taking different perspectives (knowledge access), making judgments about explicit false beliefs, and making judgments about differences between real and apparent emotions (i.e., people can feel a different emotion from the one they display). Correct responses were scaled by question difficulty (i.e., 1 point for the diverse desire question, 2 points for each of the two knowledge access questions, 3 points for each of the two false-belief questions, and 4 points for the real-apparent emotion question). A ToM score was calculated by summing correct responses for each question type such that a maximum score of fifteen could be achieved.

#### 2.2.8. Vineland Adaptive Behaviors Scales-II (VABS-II; [76])

The VABS is a semistructured parent questionnaire gauging adaptive functioning in daily living, socialization, communication, and motor skill domains. The adaptive behavior composite (ABC) is a standardized measure of adaptive behavior across the four domains.

### 2.3. Statistical Analyses

All participants provided data for at least 5 of the 7 measures. Descriptive statistics by outcome group (i.e., ASD, HR-Other, HR-TD, and LR-TD) for ADOS CSS, cognitive and affective empathy, VABS ABC, CELF CLS, ToM, observational empathy, and DAS NVIQ are reported in Table 2. The mean age of the ASD group was significantly younger than the mean ages of the other outcome groups at the time of the school-age visit (see Table 1). Therefore, age was included as a covariate in the reported analyses (ANCOVAs). Planned simple contrasts comparing ASD, HR-Other, and HR-TD to LR-TD evaluated differences among groups. ToM scores were negatively skewed and were inverse-log transformed to normalize the distribution. Subsequent tests were conducted with the transformed scores; however, to facilitate interpretation, scores reported in tables reflect original values.

In addition to examining group differences (a categorical approach), we conducted zero-order correlations among constructs related to social cognition and social communication across the entire sample (a dimensional approach). This aspect of the study aimed to quantify convergence and divergence among measures via Spearman correlations. Spearman correlations were used because some of our measures are bounded and it is a more conservative measure than Pearson correlations. Missing data were excluded pairwise to make use of all available data.

To evaluate the extent to which social-communicative, cognitive, and adaptive skills account for variability in macrolevel autism symptom severity, measures that were significantly correlated were included as predictors in a regression model using ADOS CSS as the criterion. Lastly, separate regressions (due to collinearity of the language variables) were run to evaluate whether 3-year and concurrent language skills were associated with school-age ToM. Analyses were conducted using IBM SPSS version 22 (IBM, 2013).

## 3. Results

### 3.1. Categorical Evaluations of the BAP

ANCOVAs (see Table 2) revealed group differences in ADOS CSS, ToM, core language standard score (CLS), and cognitive empathy. Group differences were not observed for the adaptive behavior composite (VABS ABC) scores, nonverbal cognitive scores (NVIQ), affective empathy, and attention to the examiner feigning distress. Planned simple contrasts indicated that both ASD and HR-Other groups had significantly higher ADOS CSS scores, and lower ToM and CLS scores than the LR-TD group. Individuals with ASD on average had significantly lower cognitive empathy scores than those in the LR-TD group. The HR-TD group did not differ significantly from the LR-TD group on any measure.

### 3.2. Dimensional Evaluations of the BAP

Spearman correlations among measures are summarized in Table 3. Nonverbal scores were only correlated with CLS and social referencing. CLS, ADOS CSS, cognitive empathy, ToM, and VABS ABC scores were intercorrelated (all *p*'s < 0.05). Cognitive empathy was not related to affective empathy. This pattern of zero-order correlations was not strongly influenced by scores from ASD participants. Associations between ADOS CSS, CLS, ToM, VABS ABC, and cognitive empathy were similar even when participants with ASD were excluded from analyses (see Table 4). Inter-related measures in the correlation matrix were entered into a regression model with ADOS CSS as the criterion. Regression diagnostics indicated that the regression model was not influenced by any outliers or influential cases [77]. ToM, VABS ABC, CLS, and cognitive empathy accounted for 27.7% of the variance in ADOS CSS scores over and above the effects of chronological age (*F*(4,46) = 4.28, *p* = 0.005). With all predictors included in the model, only ToM uniquely accounted for a significant portion of the variance in symptom severity (ToM *β* = −0.37, *t* = −2.53, *p* = 0.02, partial correlation = −0.36; VABS ABC *β* = −0.02, *t* = 0.11, and *p* = 0.91; cognitive empathy *β* = −0.13, *t* = 0.85, *p* = 0.40, CLS *β* = −0.21, *t* = −1.20, and *p* = 0.23). This finding was followed up using a holdout cross-validation method [78] to confirm the predictive validity of the regression model.

### 3.3. Predictive and Concurrent Associations between Language and ToM

Early (36-month) and school-age language scores were highly correlated with each other (*r* = 0.71, *p* < 0.001). Given excessive collinearity between the two variables, separate regressions were run to evaluate their effects on ToM, controlling for age and diagnostic classification at the school-age visit. 36-month Mullen Verbal *T* scores significantly predicted school-age ToM (*β* = 0.24, *t* = 2.21, *p* = 0.03; partial correlation = 0.27); however, school-age language (CLS) was not concurrently associated with ToM (*β* = 0.16, *t* = 1.41, *p* = 0.16; partial correlation = 0.17). The partial correlation coefficient provides an estimate of the effect size.

### 3.4. Is Early Language or Concurrent ToM More Predictive of the School-Age BAP?

When 36-month Mullen Verbal *T*-Scores were entered into the regression model predicting school-age ADOS CSS scores from all concurrent variables that were significantly associated with CSS scores (except concurrent language due to excessive collinearity with 36-month language), the model was significant (*F*(4,44) = 3.48, *p* = 0.01). Again, only ToM uniquely accounted for a significant portion of the variance in symptom severity at school age (ToM *β* = −0.368, *t* = −2.35, *p* = 0.02, and partial correlation = −0.33; VABS ABC *β* = −0.008, *t* = 0.05, and *p* = 0.96; cognitive empathy *β* = −0.23, *t* = −1.51, and *p* = 0.14; 36-month Mullen Verbal *β* = −0.09, *t* = −0.59, and *p* = 0.55). Thus, concurrent ToM was a better index of school-age BAP than early language skills.

## 4. Discussion

Converging findings from categorical and dimensional approaches to the BAP in the current study suggest that researchers should consider defining the BAP among siblings of children with ASD in terms of a range of characteristics (including atypicalities of attention, language, and mood) and possibly perceptual strengths (e.g., [38, 79]) rather than by focusing only on siblings who exhibit elevated autistic-like symptoms. This recommendation is consistent with a large body of prior work demonstrating that family members of people with ASD are more likely than others to exhibit attentional, cognitive, linguistic, mood, and personality-related atypicalities (reviewed in [41]).

### 4.1. ToM and the BAP

Reduced ToM was a characteristic of the BAP among school-aged participants in the current study when assessed categorically (i.e., ToM was a challenge for children with ASD and siblings of children with ASD who were developing atypically) and dimensionally (when related to ADOS severity scores across a combined sample of high-risk and low-risk children). In fact, associations between severity scores and a number of intercorrelated measures (i.e., ToM, adaptive skills, and language) across the full sample were driven by individual differences in ToM. This finding provides support for the compatibility of categorical and dimensional approaches to the BAP while highlighting a key social-cognitive skill that contributes to the BAP at school age. The current study extends prior evidence that ToM is an aspect of the BAP among younger [53] and older individuals [8, 33, 37–39, 54] by suggesting that ToM may be a core aspect of the BAP underlying associations between autistic-like traits and other individual differences among young children who are just beginning elementary school.

Nevertheless, the current findings are inconsistent with the only other study to examine ToM among young school age siblings of children with ASD [45] as well as other studies that did not document reduced ToM among somewhat older siblings of children with ASD [55, 80]. Studies that did not observe reduced ToM among sibs-ASD used a broadly defined high-risk versus low-risk approach to characterizing the BAP. That analytic approach may have attenuated effects as only a subset of relatives of individuals with ASD exhibit categorically distinctive aspects of the BAP (e.g., [22, 39, 81]). Similarly, the contributions of ToM to the BAP that were observed in the current study may sometimes [33] but not always [56] be observed in general population samples. Therefore, replication of the current findings is needed. Nevertheless, the current study suggests that a developmentally oriented measure of ToM, which assesses the ability to achieve different mentalizing milestones, combined with analytic approaches that focus specifically on siblings of children with ASD who are developing atypically and/or that use more dimensional analytic techniques (which are more powerful than categorical approaches; [17]) may be needed to detect ToM-related atypicalities associated with the BAP.

Indeed, a reduced capacity to understand others' mental states (at least at certain points in development and when assessed with certain tests) may be integral to the BAP and compromise the quality of interpersonal relationships. Delayed development of ToM among school-aged children with ASD is believed to underlie social immaturity and atypical peer relations [75]. These difficulties with social cognition associated with the BAP may continue to create social challenges across the lifespan [8, 82].

Future research should examine mechanisms underlying atypical social cognition associated with the BAP. Given that difficulty with emotion reading is a commonly observed characteristic of the BAP when assessed categorically among relatives of individuals with ASD (reviewed by [4]) and dimensionally among college students [12, 34, 82], difficulty interpreting emotions may contribute to atypical development of ToM in the BAP. Indeed, the Reading the Mind in the Eyes Task (one of the most widely used measures of ToM) is essentially a test of complex emotion reading. However, attention to the examiner's distress and parent-reported affective empathy were not associated with either the BAP or ToM performance in the current study. This absence of associations between aspects of affective empathy and the BAP is consistent with Smith's [65] hypothesis that affective empathy is* not* impaired in ASD. Together, these findings suggest that difficulty* deciphering* others' perspectives is an aspect of the BAP at school-age while difficulty* sharing* emotions with others is not. Difficulty deciphering one's own and others' emotions may contribute to reduced ToM at school-age. Indeed, findings from the aforementioned study that employed a combined categorical and dimensional approach to exploring the BAP suggest that difficulty reading one's own emotions (alexithymia) may be a core characteristic of the BAP that should be explored in future research [16]. Alexithymia may contribute to BAP-related difficulties with ToM as challenges reading one's own emotions likely contribute to obstacles interpreting others' emotions as well.

Future research should also examine if the family environment contributes to atypical ToM development among a subset of siblings of children with ASD. For example, the presence of an older child with ASD in the household may reduce attention (and associated opportunities to practice understanding others' minds through conversation) directed towards younger siblings. Younger siblings may also mimic behaviors indicative of atypical ToM exhibited by older siblings with ASD. Alternatively, having an older sibling whose behavior is atypical (particularly if the older child cannot explain his or her behaviors) may encourage younger siblings to work at understanding their older sibling's perspective and thus support ToM development. Prior research has demonstrated that having an older sibling* without* ASD is associated with* decreased* ToM for children with ASD, presumably because the older child may compensate for the younger child's difficulties [83]. Although complicated to assess, investigations of how sibling and parental behaviors impact the ToM development of younger siblings with and without ASD are needed.

### 4.2. Language, ToM, and the BAP

As in the broader sample of which the current participants are a subset [51], younger siblings of children with ASD who were developing atypically exhibited language difficulties relative to typically developing high-risk and low-risk children. Language was also associated dimensionally with the BAP in the current study. Consistent with a large body of research demonstrating that language is associated with ToM in typical and atypical development [59, 61], baseline correlations revealed associations between school-age structural language and parent-reported cognitive (but not affective) empathy and behavioral ToM. Nevertheless, ToM uniquely contributed to the BAP at school-age; language was no longer associated with the BAP once ToM was accounted for. In addition, concurrent language was* not* associated with ToM after controlling for age and outcome group. Therefore, difficulties deciphering other minds may be a core characteristic of the BAP at school age irrespective of concurrent language ability.

In contrast to the lack of strong associations between concurrent language and ToM at school-age, language at 36 months of age* was* predictive of subsequent ToM. This finding extends prior research demonstrating that early language skills support the development of explicit ToM reasoning among typically developing children [61] and children with autism [59, 62] by suggesting that early variations in language ability among children with and without a sibling with ASD contribute to later ToM. Longitudinal associations between early language and later ToM mirror evidence that the manifestation of core symptoms of ASD varies with age (reviewed in [84]) by suggesting that skills affected by the BAP also change with time.

While 36-month language contributed to school-age ToM, school-age ToM remained uniquely associated with school-age autistic-like symptoms even after 36-month language was accounted for in analyses. This finding supports a nonlinear interpretation of development wherein early language development gives rise to a variety of social-cognitive skills which may be used to ameliorate potential autistic-like symptoms. Future research is needed to determine whether specific difficulties (re)emerge as the children grow and encounter more challenging social environments, as suggested by Gamliel and colleagues [42].

### 4.3. Developmental Profiles of HR and LR School-Age Children

Despite our observation of significant group differences in performance on a number of measures, it is worth noting that mean scores in cognitive empathy, adaptive behaviors, and language for the HR-Other group fell well within 2 standard deviations of normed mean scores. Similarly, children in the ASD group achieved verbal and nonverbal intelligence scores that are considered “average.” Several other groups using the sibling-risk design have also reported normative cognitive skills among HR participants (e.g., [22, 85]), including a similar rate of cognitive development between HR and LR children from infancy to school age [42]. Furthermore, a recent population-based study found a positive association between common genetic risk variants for ASD and general cognitive ability [86]. Our results converge with those from other samples to suggest that familial autism may represent a particular form of ASD with high nonverbal intelligence and relatively spared adaptive and language skills (see also [87–93]). A dissociation between intelligence and social skills is commonly observed in “high-functioning” individuals with ASD (e.g., [94]) and this uneven profile may be a manifestation of BAP. Further investigation of these findings is advisable before conclusions can be drawn regarding the population of later-born siblings of children with ASD.

Participants in the current sample were at the beginning of their school years when assessed. Social and academic stressors of the school environment may have progressively disruptive effects on social-communicative and social cognitive development, especially in children with social vulnerabilities (e.g., [42, 55, 95, 96]). Increased vulnerability for social-communicative difficulties during school years may also arise from genetic factors exerting a greater influence on the phenotypic expression of highly heritable traits in later childhood (e.g., [97]). Peer relations during childhood and adolescence play a pivotal role in social affective development, including social competency and emotional understanding (for review see [98]). Adults exhibiting the BAP report reduced quality and quantity of friendships [40], alluding to a history of diminished or atypical peer relations and social networks not unlike that in ASD [99]. Poor quality of social interactions may have cascading effects on the development of ToM (e.g., [100]) and other social skills. In other words, BAP features may accrue over time due to environmental and genetic factors coupled with reduced opportunities for healthy peer interactions, presenting the possibility that high-risk children may appear more impaired than low-risk children in later years than they do at the beginning of elementary school. However, substantial evidence for resilience among the siblings of children with ASD suggests that many of those who encounter such challenges will overcome them.

Data presented in this study includes both parent interviews (VABS, Griffith Empathy) and observational assessments (ToM, CELF, DAS, ADOS, and response to distress). Our results suggest that both types of information contribute to a comprehensive profile of social cognitive and communicative behaviors associated with the BAP among school-age children. We found that ToM, cognitive empathy, adaptive functioning, and language ability collectively reflected level of autism symptom severity. Furthermore, relations across a broad range of measures of social ability were apparent when evaluated via correlations, suggesting that performance on measures of ToM, cognitive empathy, adaptive functioning, and language ability may be driven by a common underlying mechanism.

Relations among social skills, social cognition, and the BAP are also apparent in adults (e.g., [8, 13]), providing some indication of developmental stability in the observed correlations. Our pattern of results corroborates previous studies demonstrating associations between language and social cognition [57, 101–103] and adaptive social functioning and social impairments [104] in ASD. The null association between affective and cognitive components of empathy also corroborates prior work [72]. Limited evidence was obtained that behavioral ToM and cognitive empathy are indeed the same construct (as stated by [63]), as associations between ToM and parent-reported cognitive empathy were relatively weak and were no longer apparent once participants with ASD were excluded from analyses.

### 4.4. Limitations

Our manner of defining what constituted the categorical operationalization of the BAP in the current study may have impacted findings. Children in our “BAP group” were judged to have a broad range of developmental concerns including social, language, and cognitive delays, as well as disruptive attention deficits and anxiety. This heterogeneity in developmental concerns is not surprising given genetic liability: autism risk genes have been associated with social anxiety [105, 106], language impairments [30], attention-deficit/hyperactivity disorder [51], bipolar disorder, major depressive disorder, and schizophrenia [107]. Differential expression, number of risk alleles, and environmental factors may give rise to a wide range of phenotypic expression among high-risk individuals without ASD (e.g., [108, 109]). Therefore, the etiologies underlying the different developmental atypicalities in the HR-Other group were likely diverse, which coupled with small sample size limits power to detect effects.

A second limitation lies in uncertainty regarding the extent to which our high-risk sample is truly at high risk for ASD. Genetic research indicates that the presence of* de novo* copy number variants (CNVs) is greater in simplex families (those with only one child with ASD) than in multiplex families (for review see [110]). If* de novo* mutations caused ASD in some probands in the current study, younger siblings may be at lower risk for ASD than in cases where mutations or other genetic risk markers were inherited. In other words, some high-risk participants in this sample may have an older sibling with sporadic as opposed to familial autism and risk for the BAP may be reduced in cases of sporadic ASD. Efforts to examine the BAP to characterize behavioral profiles and the genetic basis of ASD would benefit from studying unaffected individuals in multiplex families. The field of genetics in ASD is progressing at a rapid rate to include repositories of genetic information from families in multiple sites (e.g., Autism Genetic Resource Exchange, MSSNG, Simons Simplex Collection). Comparable behavioral databases will also move the field forward.

The small number of high- and low-risk participants with ASD and “other outcomes" in the current sample did not permit us to examine whether categorical differences between groups and dimensional associations among variables within each group index convergent aspects of the BAP. Attrition is inevitable in longitudinal research and this was a limiting factor in this study. Future research following high- and low-risk participants through and beyond toddlerhood would benefit from multisite collaboration. This would both increase power to detect effects and improve generalizability of results.

## 5. Conclusions and Future Directions

The current study provides support for the compatibility of categorical and dimensional approaches to the BAP and suggests that ToM may be a key aspect of the BAP among children with a sibling with ASD who are just starting out in school. Therefore, supports for siblings of children with ASD who are preparing to enter school should focus proactively on helping them develop ToM skills. Reading has been associated with ToM development among children [111]. Narrative-based approaches to promoting ToM, such as reading groups where children practice reflecting on and telling stories, may be beneficial for preschool and school-age siblings of children with ASD. Literacy-based ToM supports may also help to avoid the potential exacerbation of language difficulties among younger siblings as they develop and encounter more challenging environments.

Greater understanding of potential precursors to ToM may yield insights into the development of social functioning and the emergence of the BAP. One theoretical precursor to ToM is joint attention (e.g., [112]). Joint attention is believed to be crucial for social cognitive development, language acquisition, and social competence (for a review, see [113]). Furthermore, difficulties with joint attention are often (but not always) an aspect of the BAP in infancy [114–117]. Empirically evaluating a relation between early joint attention skills and subsequent ToM reasoning among high- and low-risk toddlers would be a promising future endeavor.

## Figures and Tables

**Table 1:** Participant characteristics by group.

	HR-ASD (*n* = 11)	HR-Other (*n* = 12)	HR-Typical (*n* = 24)	LR-Typical (*n* = 22)	*p* ^1^
Male, %	63.64	50.00	37.50	59.09	0.39
Caucasian, %	36	31	58	84	0.001
High SES^2^, %	60	50	24	52	0.25
Age at testing in years, M (SD)	5.46 (0.46)	6.04 (1.29)	5.52 (0.45)	5.89 (0.41)	0.01

*Note. *Some families did not provide complete demographic data. Percentages are relative to all responses received.

^1^ *p* value = significance level for Kruskal-Wallis test of overall group differences.

^2^High SES = annual family income ≥ USD $125,000.

**Table 2:** Descriptive statistics by group.

	ASD	HR-Other	HR-TD	LR-TD	*F*	*p*	*η* ^2^
ADOS CSS	7.18^a^ (0.98)	5.00^b^ (2.63)	1.75 (0.94)	1.59 (1.18)	50.66	<0.001	0.70
ToM scaled score	6.67^c^ (3.17)	9.09^d^ (3.65)	11.13 (3.72)	12.36 (2.85)	7.84	<0.001	0.27
CELF-4 Core Language SS	90.00^c^ (22.23)	93.17^d^ (18.82)	108.04 (11.95)	109.73 (10.65)	7.76	<0.001	0.27
Griffith cognitive empathy	0.63^c^ (5.73)	6.25 (8.61)	9.55 (7.39)	8.84 (6.29)	4.23	0.009	0.19
VABS ABC	86.82 (15.17)	93.63 (18.95)	102.09 (25.62)	105.87 (12.41)	2.41	0.08	0.12
DAS NVIQ	105.27 (12.91)	104.92 (16.67)	107.71 (13.43)	104.32 (9.66)	0.17	0.92	0.08
Social reference to examiner (%time)	76.63 (31.81)	79.10 (30.31)	69.75 (32.77)	72.00 (25.18)	0.69	0.56	0.03
Griffith affective empathy	6.13 (9.30)	9.67 (8.18)	9.64 (7.56)	6.42 (11.29)	0.612	0.61	0.03

*Note*. Standard deviations appear in parentheses below means. Analyses of variance were covaried by age.

^a^ASD > LR-TD; ^b^HR-Other > LR-TD; ^c^ASD < LR-TD; ^d^HR-Other < LR-TD.

**Table 3:** Behavioral intercorrelations.

	Variable	1	2	3	4	5	6	7
1	DAS NVIQ							
2	CELF-4 Core Language SS	0.28^a^						
3	ADOS CSS	−0.02	−0.42^b^					
4	Griffith affective empathy	0.01	−0.08	−0.14				
5	Griffith cognitive empathy	−0.04	0.30^a^	−0.24	0.11			
6	Social reference %	0.30^a^	−0.15	0.03	0.14	−0.001		
7	ToM scaled score	0.09	0.40^b^	−0.46^b^	−0.17	0.25^a^	0.07	
8	VABS ABC	0.21	0.55^b^	−0.35^a^	−0.13	0.51^b^	−0.08	0.44^b^

*Note.* ^a^ *p* < 0.05; ^b^ *p* < 0.01.

**Table 4:** Behavioral intercorrelations, excluding ASD participants.

	Variable	1	2	3	4	5	6	7
1	DAS NVIQ							
2	CELF-4 Core Language SS	0.38^b^						
3	ADOS CSS	−0.04	−0.32^a^					
4	Griffith affective empathy	0.02	−0.05	−0.13				
5	Griffith cognitive empathy	0.02	0.26	−0.09	0.04			
6	Social reference %	0.23	−0.08	0.02	0.17	0.05		
7	ToM scaled score	0.07	0.33^b^	−0.30^a^	−0.23	0.15	0.09	
8	VABS ABC	0.35^a^	0.34^a^	−0.11	−0.12	0.42^b^	0.07	0.33^a^

*Note.* ^a^ *p* < 0.05; ^b^ *p* < 0.01.
